# Structural Analysis of Soluble Elastin in Dry and Hydrated States Using ^13^C Solid-State NMR

**DOI:** 10.3390/polym17192638

**Published:** 2025-09-30

**Authors:** Tetsuo Asakura, Akira Naito, Keiichi Miyamoto

**Affiliations:** 1 Department of Biotechnology, Tokyo University of Agriculture and Technology, Koganei 184-8588, Japan; 2 Division of Applied Chemistry, Graduate School of Engineering, Mie University, Tsu 514-8507, Japan

**Keywords:** elastin, ^13^C solid-state NMR, alanine, α-helix, random coil, cross-linking

## Abstract

Elastin is the principal protein found in the elastic fibers of vertebrate tissues, and the water within these fibers plays a crucial role in preserving the structure and function of this hydrophobic protein. Soluble elastin was successfully obtained by repeatedly treating insoluble elastin, extracted from pig aorta, with oxalic acid. Solid-state NMR analysis was performed on the soluble elastin, focusing on conformation-dependent chemical shifts of alanine residues. This analysis revealed that cross-linked alanine residues exhibited both α-helix and random coil structures in the dry state. In contrast, the hydrated state favored random coil structures, with some distorted helices possibly present, indicating that the cross-linked configuration is relatively unstable. Similar conformational changes were observed in insoluble elastin, mirroring those found in the soluble form. Additionally, when the soluble elastin was re-cross-linked using 1,12-dodecanedicarboxylic acid and 4-hydroxyphenyl dimethylsulfonium methylsulfate, it retained a mixture of α-helix and random coil structures in the dry state. Remarkably, in the hydrated state, α-helix structures were more prominently preserved alongside random coils. These structural changes corresponded with increased stiffness of molecular chains in the hydrophobic regions compared to their state prior to re-cross-linking, even under hydrated conditions.

## 1. Introduction

Elastic fibers play a crucial role in providing flexibility and durability to mammalian tissues like blood vessels and lungs. These mechanical properties largely stem from a specialized protein known as elastin [1,2]. Elastin is an insoluble, extensively cross-linked protein that originates from a soluble precursor called tropoelastin, which has a molecular weight ranging from 60 to 70 kDa. Tropoelastin is composed of multiple domains that alternate between hydrophobic segments and cross-linking regions. The hydrophobic portions are abundant in nonpolar amino acids such as proline (Pro:P), glycine (Gly:G), valine (Val:V), and leucine (Leu:L). The function of elastin is to endow biological tissues with elasticity, and the elasticity of elastin is achieved only when tropoelastin chains are polymerized and swollen in water [3]. Experimental data and molecular dynamics simulations indicate that these hydrophobic domains are highly flexible and lack a rigid structure in solution [4,5,6,7,8,9]. Their disordered nature and hydrophobicity are believed to contribute significantly to the elasticity and stretchability of the elastin polymer [10,11,12]. In contrast, the cross-linking domains are rich in alanine (Ala:A) and lysine (Lys:K), and they stabilize the elastin network through covalent bonds formed by lysine side chains. Sequences like KAAK or KAAAK, which feature polyalanine stretches interrupted by lysine residues, are thought to adopt α-helical conformations. This structural arrangement may position lysine residues on the same side of the helix, facilitating efficient cross-link formation [7,13,14,15,16,17,18].

To explain the reversible stretching and recoiling behavior of elastic fibers, researchers have proposed a variety of structural models over the years [19,20,21,22]. One of the most widely supported hypotheses posits that elastin exists in a dynamic balance between structured secondary elements and disordered random coil configurations [7,22]. This idea—often described as a “conformational ensemble” [7] or “structural distribution” [23,24,25]—is backed by findings from Fourier transform infrared (FTIR) and circular dichroism (CD) spectroscopy, particularly studies involving elastin-like peptides [7,26,27,28]. Among the secondary structures that may arise in elastin’s hydrophobic domains, the β-turn stands out as especially prevalent [29]. To account for elastin’s elastic properties, especially in proline-rich sequences like VPGVG, the “sliding β-turn” model has been proposed [22,30,31,32]. To investigate these structural models in greater detail, researchers have employed solid-state nuclear magnetic resonance (NMR) spectroscopy, which has provided valuable insights across numerous studies [4,17,18,23,24,25,33,34,35,36,37,38,39,40].

Although elastin exhibits excellent mechanical characteristics, its application in biomaterials has been significantly constrained due to its high level of insolubility, which stems from extensive intermolecular cross-linking. To obtain soluble elastin, hydrolytic treatment is commonly used to break down its native, insoluble form. In its natural state, elastin undergoes enzymatic cross-linking that leads to the production of desmosine, isodesmosine, and similar compounds [41]. These cross-linked structures are crucial for maintaining both the mechanical strength and biological functionality of elastin. To recover its performance, chemical re-crosslinking approaches have been investigated, utilizing agents such as 1,12-dodecanedicarboxylic acid (Dode) and 4-hydroxyphenyl dimethylsulfonium methylsulfate (DSP) [42]. These reagents assist in reconstructing the necessary structural attributes that enable elastin to function effectively in biomedical applications.

In earlier work, Miyamoto and collaborators isolated insoluble elastin from porcine arterial tissues, successfully retaining its hydrophobic segments and native cross-linked architecture. To produce a soluble variant of elastin, they employed the protocol introduced by Partridge and co-workers [43,44], which involves repeated exposure of insoluble elastin to oxalic acid, followed by cooling and centrifugation to obtain a yellow-colored solution. After approximately eight cycles of this process, the elastin was fully solubilized. At the same time, to investigate the structural and dynamic properties of biological specimens under both dry and hydrated conditions, a powerful strategy has emerged that integrates three solid-state NMR methodologies: ^13^C refocused INEPT (Insensitive Nuclei Enhanced by Polarization Transfer), ^13^C CP/MAS (Cross Polarization with Magic Angle Spinning), and ^13^C DD/MAS (Direct Polarization with Dipolar Decoupling and Magic Angle Spinning) [45,46,47]. Among these, ^13^C INEPT—originally designed for solution-state NMR—is particularly responsive to fast-moving segments in hydrated proteins. In contrast, ^13^C CP/MAS is selective for rigid, slowly moving regions, and its signal intensity diminishes as hydration increases molecular mobility, making such regions less detectable. Meanwhile, ^13^C DD/MAS is capable of identifying both flexible and rigid domains, offering a more complete picture of molecular structure and motion [48].

The aim of the present study is to investigate the structural differences of alanine residues in the cross-linked regions of soluble elastin—developed by Miyamoto et al. and expected to be highly promising as a future biomaterial—in both dry and hydrated states using three types of solid-state NMR: ^13^C INEPT, ^13^C CP/MAS, and ^13^C DD/MAS. Additionally, the study evaluates the structural effects of re-crosslinking soluble elastin with chemical crosslinkers Dode and DSP [49] by examining the re-crosslinked elastin in both dry and hydrated states using the same three solid-state NMR techniques.

## 2. Materials and Methods

### 2.1. Preparation of Insoluble Elastin

Porcine arteries were sourced from a slaughterhouse located in Matsusaka, Mie, Japan [42]. After trimming away excess fat from approximately 2 kg of arterial tissue using scissors, the samples were immersed in a 10 wt% sodium chloride solution and stored at 4 °C for 24 h. The tissue was then chopped into 5 mm fragments using a blender and subjected to autoclaving at 121 °C for one hour. Following this, the fragments were rinsed with deionized water and soaked in 90 wt% ethanol for 10 h. Once dried and ground, a total of 330 g of insoluble elastin powder was successfully produced.

### 2.2. Preparation of Soluble Elastin

Soluble elastin was extracted from insoluble elastin following the procedure developed by Partridge et al. [43,44]. In summary, 10 g of insoluble elastin was combined with 45 mL of 0.25 M oxalic acid and heated at 100 °C for one hour. The resulting transparent yellow solution was then cooled to 4 °C and centrifuged at 2000× *g* for 30 min. The undissolved residue was treated again with 30 mL of 0.25 M oxalic acid under the same heating conditions. The portion solubilized during the initial oxalic acid treatment was designated as Soluble Elastin (1). This extraction process was repeated a total of eight times until the elastin was fully dissolved, with the final product labeled as Soluble Elastin (8). The collected solution was dialyzed against deionized water for seven days at 4 °C using a cellulose acetate membrane (molecular weight cutoff: 12–16 kDa; Sanko Junyaku Co., Ltd., Tokyo, Japan). The soluble elastin was then recovered as a dry powder through freeze-drying. For this study, the fraction obtained from the eighth solubilization step was used as Soluble Elastin (8).

### 2.3. Molecular Weight Analysis and Amino Acid Analyses

The molecular weight of soluble elastin was assessed via gel permeation chromatography (GPC) employing a TSK-gel G3000SW column (Tosoh Corp., Tokyo, Japan). A 0.1 M phosphate-buffered saline (PBS, pH 7.0) containing 0.1 M NaCl served as the elution buffer, with a flow rate maintained at 0.5 mL/h and a column temperature of 10 °C. A sample volume of 20 μL (concentration: 10 mg/mL) was injected, and detection was performed at 280 nm using a UV spectrophotometer (Hitachi, Tokyo, Japan). Molecular weight estimation was carried out by referencing calibration standards ranging from 1.2 to 670 kDa (Bio-Rad Laboratories, Hercules, CA, USA). The GPC results suggest that low-molecular-weight fragments under approximately 5 kDa were effectively eliminated through dialysis using a cellulose acetate membrane.

Amino acid composition of the soluble elastin was determined using an automated amino acid analyzer (JLC/500V; JEOL Ltd., Tokyo, Japan). Prior to analysis, samples underwent hydrolysis in 6 N hydrochloric acid at 110 °C for 24 h under vacuum conditions.

### 2.4. Cross-Linking of Soluble Elastin Using Dode-DSP

The powdered water-soluble elastin and Dode-DSP (Extracellular Matrix Laboratories, Tsu, Japan) were dissolved in Milli-Q water to make a 40% aqueous solution. After stirring, the pre-gel solution was incubated at 70 °C for 30 min to allow coacervation, followed by incubation at 100 °C for 60 min to allow gelation. After gelation, the samples were washed with deionized water for 18 h and freeze-dried to obtain the samples. The Dode-DSP crosslinker ratio was expressed as x1 when it was equal to the total mol of amino groups obtained from the amino acid analysis of soluble elastin. The samples were prepared by varying the Dode-DSP crosslinker ratio (×1, ×3, ×5, and ×7).

### 2.5. Preparation of (Val-Pro-Gly-Val-Gly)_6_

(VPGVG)_6_ sample was synthesized by the solid-phase Fmoc chemistry on a fully automated Pioneer Peptide Synthesis System (Applied Biosystems Ltd., Waltham, MA, USA). The sample was purified by HPLC using acetonitrile as an eluent. After purification, acetonitrile was removed, and then the aqueous solution was lyophilized [24].

### 2.6. NMR Measurements

Solid-state ^13^C NMR spectra for all samples were acquired using either a Bruker Avance 400 or JEOL ECX 400 spectrometer (Billerica, MA, USA), both equipped with a 4 mm double-resonance MAS probe. Measurements were conducted at 25 °C with a spinning rate of 8.5 kHz. For hydrated soluble elastin samples, a zirconia rotor sealed with a PTFE insert was used to maintain full hydration after treatment. The water content was 65 wt% for insoluble elastin and 60 wt% for crosslinked soluble elastin at a concentration of 40 wt%, ensuring sufficient hydration in both cases. Typical parameters for ^13^C CP/MAS NMR experiments included a 3.5 μs 90° pulse for ^1^H, a 1 ms ramped cross-polarization pulse at 71.4 kHz RF field strength, TPPM ^1^H decoupling during acquisition, 2048 data points, 8000 scans, a spectral width of 310 ppm, and a recycle delay of 4 s. Conditions for ^13^C DD/MAS NMR experiments followed those reported previously [48], with 8000 scans, a 5 s recycle delay, and a 3.5 μs 90° pulse for ^13^C. Refocused INEPT experiments employed a 3.5 μs pulse for ^1^H and a 3.6 μs pulse for ^13^C, with an inter-pulse delay of 1/4^1^J_CH_ (^1^J_CH_ = 145 Hz). Refocusing delays were set to either1/6^1^J_CH_ or 1/3^1^J_CH_. TPPM ^1^H decoupling was applied during acquisition, with 1438 data points, 4000 scans, a 200 ppm sweep width, and a 3.5 s recycle delay. ^13^C chemical shifts were externally referenced using the methylene signal of adamantane at 28.8 ppm, relative to TMS at 0 ppm. Solution-state ^13^C NMR of soluble elastin was performed using the DEPT technique on a JEOL ECX 400 spectrometer, with ^1^H irradiation. The delay time was set to 2 s, the flip angle was 30°, and 3500 scans were collected. The sample concentration in aqueous solution was 10 wt%.

## 3. Results and Discussion

### 3.1. Amino Acid Sequence of Tropoelastin and Amino Acid Composition of Soluble Elastin

**Figure 1:**
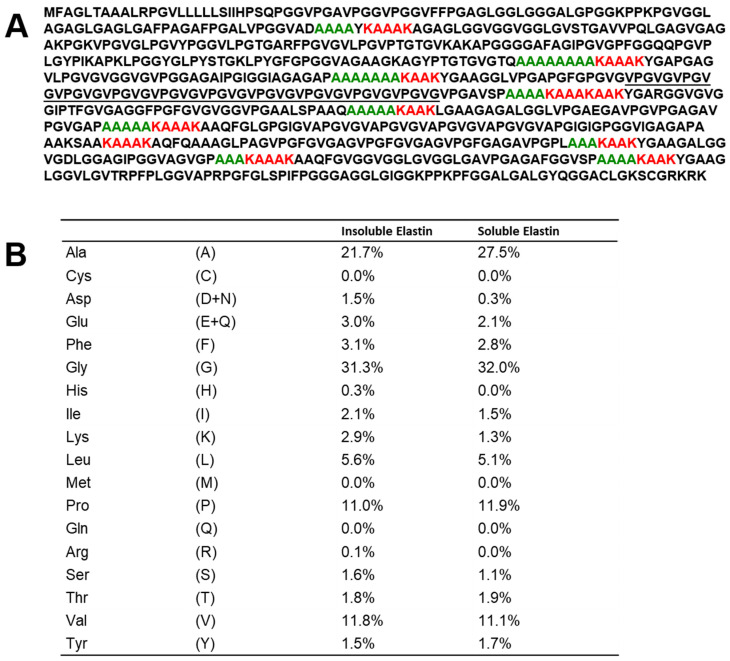
(**A**). Amino acid sequence of tropoelastin from porcine arteries (GenBank:BAP76076.1) and (**B**). Amino acid compositions of insoluble and soluble elastins.

The amino acid sequence of tropoelastin from porcine arteries obtained from GenBank:BAP76076.1 was shown in Figure 1A. The red characters of KAAK, KAAAK, and KAAAKAAK are considered to be cross-linked sequences and are possible to form the α-helix (a) [15]. The green characters of polyalanine sequences before the cross-linking part are also possible to form α-helical part of elastin. There are many PG sequences together with GG sequences. Especially, the repeated sequences, (VPGVG)_10_ underlined, are the most famous sequences in hydrophobic regions of elastin, and the structure and dynamics have been studied in detail [21,22,23,24,50,51,52,53,54]. Miyamoto et al. [42] repeatedly treated insoluble elastin with oxalic acid, followed by cooling and centrifugation of the resulting yellow solution. The cycle was repeated, and the digests obtained from dissolutions were used as soluble elastin (Soluble Elastin (8): Mw (Weight Average Molecular Weight) = 90.3 KDa, Mn (Number Average Molecular Weight) = 25.6 KDa, Mw/Mn = 3.5. Figure 1B is the amino acid compositions of insoluble and soluble elastins prepared here, and we have come to realize that their compositions bear a considerable resemblance to each other, although the fraction of Ala residue increased slightly in soluble elastin. The concentration of Ala is thought to result from the selective removal associated with hydrolysis by oxalic acid in hydrophobic regions. In particular, the hydrophobic regions near the cross-linking sites where desmosine is formed are considered highly susceptible to hydrolysis. The amino acid composition is rich in hydrophobic amino acids, with Gly, Ala, Val, Pro, and Leu being typical examples. These amino acids account for more than 80% of the total composition of elastin.

### 3.2. Peak Assignments of ^13^C Solid-State NMR Spectra of Soluble Elastin in the Dry and Hydrated States

The function of elastin is to endow biological tissues with elasticity, and elastin exhibits its flexible properties only when tropoelastin, a soluble precursor, chains are polymerized and hydrated; otherwise, dry elastin is brittle. Tropoelastin undergoes assembly wherein lysine side chains are chemically modified to create bifunctional crosslinks such as allysine aldol and dehydrolysinonorleucine, alongside tetrafunctional structures like desmosine and isodesmosine [41]. These extensive crosslinks contribute significantly to elastin’s insolubility. Combined with its hydrophobic nature and high molecular weight, these characteristics limit the use of common structural analysis methods, such as X-ray crystallography and solution-state NMR spectroscopy. As a result, solid-state NMR, supported by conformation-sensitive NMR chemical shifts, has proven effective. Therefore, we employed three types of solid-state NMR techniques for structural analysis of hydrated elastin samples: INEPT (sensitive to rapidly moving segments), CP/MAS (sensitive to slowly moving segments), and DD/MAS (sensitive to both mobile and immobile segments). Additionally, CP/MAS was used to analyze the dried elastin samples [45,46,47,48,55,56].

**Figure 2:**
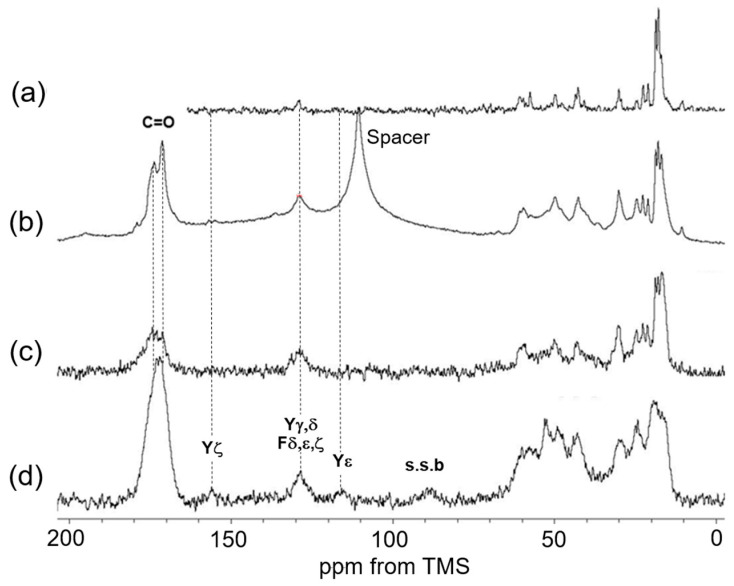
Stacked ^13^C solid-state NMR spectra (0–200 ppm) of soluble elastin. (**a**) ^13^C INEPT NMR, (**b**) ^13^C DD/MAS NMR, and (**c**) ^13^C CP/MAS NMR spectra in the hydrated state. (**d**) ^13^C CP/MAS spectrum in the dry state.

Figure 2 presents the ^13^C solid-state NMR spectra (0–200 ppm) of soluble elastin under various conditions: (a) ^13^C INEPT spectrum, (b) ^13^C DD/MAS spectrum, (c) ^13^C CP/MAS spectrum in the hydrated states, and (d) ^13^C CP/MAS spectrum in the dry state. In all spectra except the INEPT spectrum, peaks corresponding to the aromatic carbons of phenylalanine (Phe:F) and tyrosine (Tyr:Y) residues, as well as carbonyl carbon peaks, were observed. The sharp, isolated peaks in the ^13^C INEPT spectrum were assigned to ^13^C random coil (r.c.) peaks exhibiting rapid molecular motion in the hydrated soluble elastin powder. Notably, no peaks appear in the carbonyl region of the INEPT spectrum, as carbonyl carbons lack directly bonded ^1^H nuclei, which are required for INEPT detection. In the DD/MAS NMR spectrum, a rotor-synchronized peak was observed at 110 ppm. Additionally, a spinning sideband peak of carbonyl carbon at 89 ppm was detected in the ^13^C CP/MAS spectrum of the dry sample. The ^13^C CP/MAS spectrum in the dry state exhibited broad peaks, indicative of ^13^C nuclei undergoing slow molecular motion. To facilitate further peak assignments and spectral comparisons among the three hydrated samples and the dry sample, expanded spectral regions (0–80 ppm) of the ^13^C INEPT, ^13^C DD/MAS, and ^13^C CP/MAS spectra in the hydrated state, along with the ^13^C CP/MAS spectrum in the dry state, are presented as follows.

**Figure 3:**
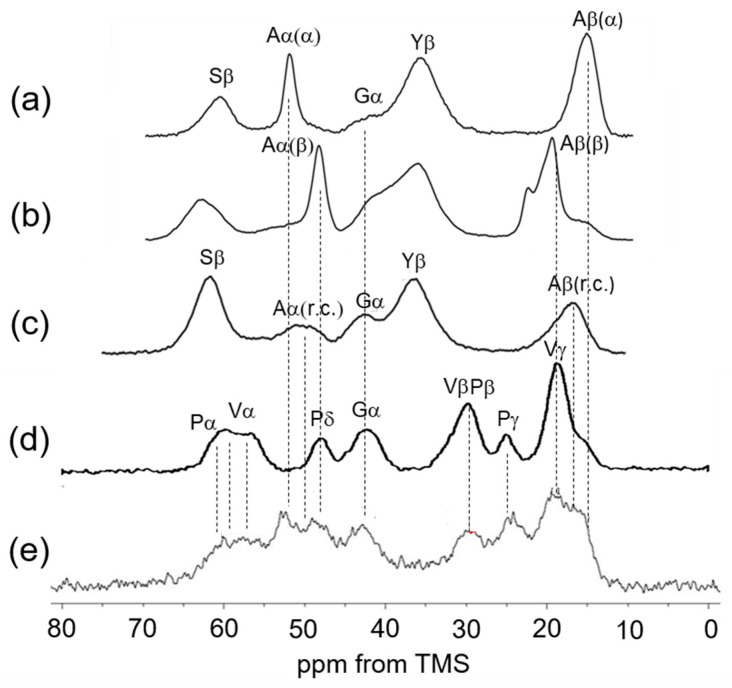
(**a**) ^13^C CP/MAS NMR spectra of [3-^13^C]Ser, [3-^13^C]Tyr and [3-^13^C]Ala-labeled *S. c. ricini* silk fibroin (**a**) before spinning and (**b**) after spinning in the dry states together with (**c**) that of [3-^13^C]Ser, [3-^13^C]Tyr and [3-^13^C]Ala-labeled *B. mori* silk fibroin sponge with amorphous form in the dry state. (**d**) ^13^C CP/MAS NMR spectrum of (Val-Pro-Gly-Val-Gly)_6_ in the dry state together with (**e**) ^13^C CP/MAS NMR spectrum of soluble elastin in the dry state.

Figure 3 shows the ^13^C CP/MAS NMR spectra of [3-^13^C]Ser, [3-^13^C]Tyr, and [3-^13^C]Ala-labeled *Samia cynthia ricini* (*S. c. ricini*) silk fibroin in the dry state: (a) before spinning and (b) after spinning [55]. These are presented alongside (c) the spectrum of [3-^13^C]Ser, [3-^13^C]Tyr, and [3-^13^C]Ala-labeled *Bombyx mori* (*B. mori*) silk fibroin sponge, (d) the ^13^C CP/MAS NMR spectrum of (VPGVG)_6_ in the dry state, and (e) the ^13^C CP/MAS NMR spectrum of soluble elastin in the dry state. For the assignment of the Ala Cα and Cβ peaks of Figure 3e, previous assignments from the ^13^C CP/MAS NMR spectra of *S.c. ricini* silk fibroin [55] and *B. mori* silk fibroin [56] were referenced. Since the amino acid composition of elastin is rich in Gly and Ala, similar to silk fibroin, it is considered appropriate to use the NMR peak assignments of silk fibroin as a reference for elastin. *S.c. ricini* silk fibroin consists of alternating Ala-rich regions, typically containing 12 or 13 consecutive Ala residues, and Gly-rich regions. When the silk fibroin stored in the silk gland is extracted and dried under mild conditions, its structure is known to adopt a typical α-helix conformation, as shown in Figure 3a [55]. In contrast, after fiber formation, the structure transitions to a typical β-sheet (Figure 3b). *B. mori* silk fibroin consists of repeated AGAGSG sequences and Gly-rich regions. When regenerated silk fibroin is dissolved in water and freeze-dried, its structure is known to adopt a random coil conformation, as shown in Figure 3c [56]. Therefore, the chemical shift references for Ala Cα and Cβ carbons of soluble elastin in α-helix, β-sheet, and random coil conformations can be obtained from the ^13^C CP/MAS NMR spectra of these silk fibroins in the dry state.

For assignments of Gly, Val, and Pro carbons, the chemical shifts of these carbons of the ^13^C CP/MAS NMR spectrum of (VPGVG)_6_ in the dry state are used [24]. The repeated sequences of (VPGVG) appeared frequently in the amino acid sequence of elastin, as shown in Figure 1A. The characteristic chemical shifts are as follows: Val Cγ at 18–19 ppm, Pro Cγ at 24.6 ppm, Gly Cα at 42.6 ppm, Pro Cδ at 48.4 ppm, and Val Cα and Pro Cα between 57 and 61 ppm [34,37]. The Val Cα peak appears broader, with the explanation to follow later. The assignment of Ala peaks is relatively complex. However, the Ala Cα peak at 52.5 ppm in the ^13^C CP/MAS NMR spectrum of soluble elastin is clearly attributed to the Cα carbon of Ala residues in an α-helix structure, based on comparison with the spectrum of *S. c. ricini* silk fibroin before spinning.

**Figure 4:**
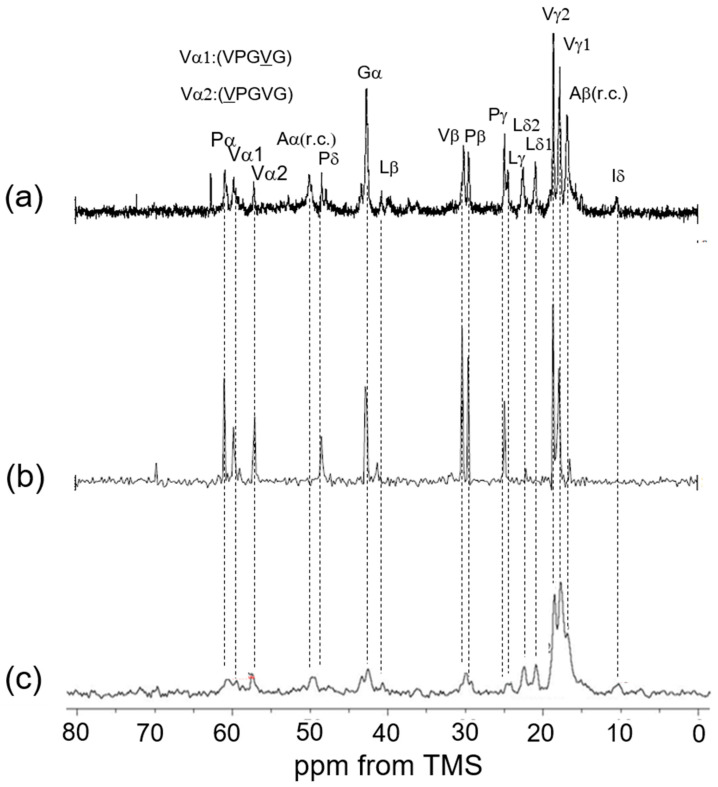
(**a**) ^13^C solution NMR spectrum of soluble elastin in aqueous solution. (**b**) ^13^C INEPT spectrum of (Val-Pro-Gly-Val-Gly)_6_ in the hydrated state, and (**c**) ^13^C INEPT spectrum of soluble elastin in the hydrated state.

Among the Cβ peaks ranging from 14 to 20 ppm in soluble elastin, the 15 ppm peak, appearing as a shoulder of the broad peak in this range, is assigned to the Ala Cβ in the α-helix structure of *S. c. ricini* silk fibroin. The broad Ala Cβ peak at 16.6 ppm, attributed to the random coil in soluble elastin, is also believed to be present within the broad peak ranging from 14 to 20 ppm, as observed in the spectrum of [3-^13^C]Ser-, [3-^13^C]Tyr-, and [3-^13^C]Ala-labeled *B. mori* silk fibroin sponge. Consequently, the broad Ala Cα peak around 50 ppm, assigned to the random coil conformation, is likewise expected to be a part of the overlapping peak in the 46–53 ppm range. The presence of these random coil Ala peaks is described in the INEPT spectrum later. A key issue is whether β-sheet structures are present in soluble elastin. Previous studies have denied the existence of β-sheet structure in elastin before the coacervate state [17,18,27,34]. Since the observed soluble elastin sample was in a fully hydrated state (100%) [33] and at an NMR observation temperature of 25 °C, coacervation did not occur [42]. Figure 4 shows (a) the ^13^C solution NMR spectrum of soluble elastin in aqueous solution and the ^13^C INEPT spectra of (b) (VPGVG)_6_ and (c) soluble elastin in the hydrated state. The mobility of the molecular chain increases in aqueous solution, the peaks become sharper, and the spectral resolution improves. As a result, several peaks could be observed as the two split peaks, i.e., Val γ_1,2_, Leu δ_1,2_, Pγ, and Lγ, Vβ, and Pβ, Vα_1_ (VPGVG), and Vα_2_ (VPGVG) in Figure 4a, although these peaks were observed as broad single peaks in the ^13^C CP/MAS NMR spectra in the dry state, as shown in Figure 3e. Here, the chemical shift of the Val Cα peak differs by 2.7 ppm between different conformations, (VPGVG)_6_ and (VPGVG)_6_. This variation is attributed to steric interactions between the Val side chain and the pyrrolidine ring of the adjacent Pro residue, which restricts the conformational distribution of Pro following Val residues [24,57]. The two split peaks are also maintained in the case of the INEPT spectrum of (VPGVG)_6_. Thus, the spectral assignment of soluble elastin in the ^13^C INEPT spectrum (Figure 4c) could be performed using these assignments, as shown in Figure 4a. The fraction of Ala residues is considerably lower than the expected value based on the amino acid composition of the mobile region shown in Figure 1B. This is attributed to the contribution of Ala residues in the formation of cross-linking. Furthermore, the relative intensities of Val, Pro, and Leu carbons are reduced in the INEPT spectrum, suggesting that the backbone of the hydrophobic domain is stiffer. The Ala Cβ random coil peak was observed at 16.6 ppm together with the Ala Cα random coil peak at 50.0 ppm in the INEPT spectrum.

### 3.3. ^13^C Solid-State NMR Spectra of Soluble Elastin in the Dry and Hydrated States

**Figure 5:**
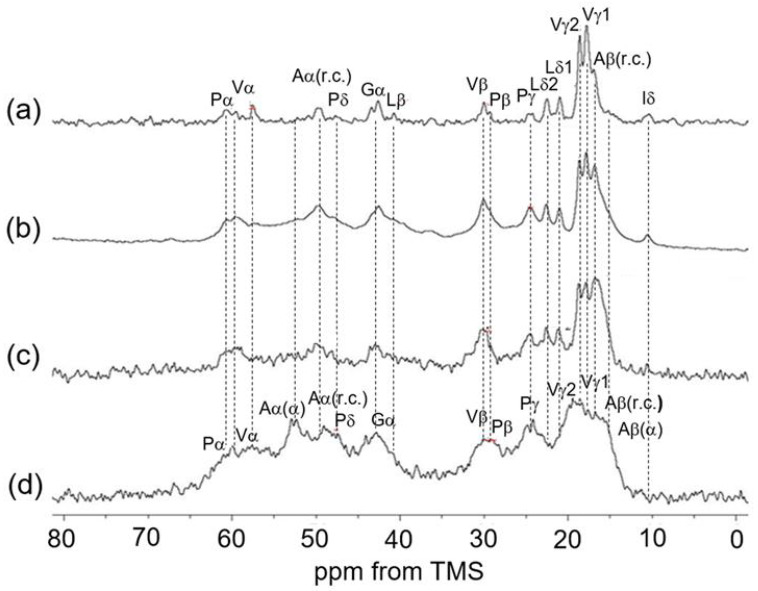
A series of ^13^C solid-state NMR spectra (0–80 ppm) of soluble elastin together with the assignment: (**a**) ^13^C INEPT spectrum, (**b**) ^13^C DD/MAS spectrum, and (**c**) ^13^C CP/MAS spectrum in the hydrated states. (**d**) ^13^C CP/MAS spectrum in the dry state.

Figure 5 summarizes a series of ^13^C solid-state NMR spectra (0–80 ppm) of soluble elastin together with the assignment: (a) ^13^C INEPT spectrum, (b) ^13^C DD/MAS spectrum, (c) ^13^C CP/MAS spectrum in the hydrated state, and (d) ^13^C CP/MAS spectrum in the dry state. By comparing the ^13^C CP/MAS NMR spectra in the dry and hydrated states, a remarkable change was observed: the Ala Cα helical peak at 52.5 ppm, clearly visible in the dry state (Figure 5d), almost disappeared in the ^13^C CP/MAS NMR spectrum of the hydrated state. Similarly, the Ala Cβ helical peak, prominent in the dry state, diminished significantly in the hydrated state. If the Ala residues retained their helical states while exhibiting increased mobility, the Ala Cα peak would not be visible in the ^13^C CP/MAS NMR spectrum under hydrated conditions. However, it should appear in the hydrated state INEPT spectrum. Nevertheless, the Ala Cα helical peak at 52.5 ppm is not observed in the INEPT spectrum. Therefore, it is considered that the Ala residues, which were in a helical state under dry conditions, have transitioned to a random coil under hydrated conditions. However, small peaks observed between the α-helix (15 ppm) and random coil (16.6 ppm) regions in the Ala Cβ peak suggest the possible presence of a distorted helix, even though the main peak corresponds to a random coil. Therefore, we attempted spectral deconvolution to determine the proportion of each conformation. However, the spectrum was unfortunately too broad and severely overlapping, making this impossible. There are reports that increasing the measurement temperature can sharpen the spectrum [34,37], but we did not pursue this approach due to the possibility of coacervation occurring. In any case, the Ala residues located in the helical state near the terminal region of the cross-linked domain are thought to have unraveled into a random coil or a partly distorted helix due to the presence of water. This means that the α-helix structures of Ala residues of soluble elastin in the dry state are unstable, and water changes the α-helix structure easily.

Ala residues are frequently found in elastin’s tandem repeats, such as (APGVGV)_3_ [58] and KAAK, KAAAK, and KAAAKAAK, as well as the polyalanine sequences prior to cross-linking [7,15,59,60]. Thus far, KA-type cross-linking domains are assumed to be mostly α-helical structures, but the α-helical content observed by CD or solid-state NMR in the hydrated state was considerably lower than would be expected for a fully formed helical structure [7,15,16,18,27,61,62]. To assess the stability of secondary structures, the CD spectra of full-length human tropoelastin were examined across rising temperatures [27]. The absorbance at 222 nm and 202 nm diminished progressively with the temperature increase, highlighting a reduction in secondary structure within both hydrophobic and cross-linking domains. Notably, the absorbance at 222 nm had almost completely faded by 37 °C, indicating that the α-helical structure of tropoelastin becomes unstable at and above physiological temperatures. These observations suggest that, under normal physiological conditions, the α-helical structure in the cross-linking domains of tropoelastin is highly unstable. In this experiment, Ala residues, which were in an α-helical state under dry conditions, have transitioned to mainly a random coil and partially distorted helical structures under hydrated conditions. Kumashiro et al. [33] reported significant spectral changes in elastin from bovine nuchal ligament in the ^13^C CP/MAS NMR spectra under dry and hydrated conditions, as well as with temperature variations, although the chemical shift changes from α-helix to random coil in Ala residues were not highlighted. Djajamuliadi et al. [17,18] prepared [U-^13^C]Ala-elastin through neonatal rat smooth muscle cell culture and analyzed hydrated [U-^13^C]Ala-elastin at 37 °C using ^13^C INEPT, DD/MAS, and CP/MAS NMR spectroscopies. They concluded that Ala residues within hydrophobic domains are predominantly in a random coil conformation, with a minor presence of distorted-helical structures. Meanwhile, the cross-linking domains exhibited a mixture of helices and random coil structures—a conclusion basically consistent with our findings, although the Ala structure could not be separated into a hydrophobic domain and a cross-linking domain in our study.

In our investigation of hydrophobic regions, we observed that the chemical shifts of Gly, Val, Pro, and Leu residues remained consistent between dried and hydrated elastin samples. These non-polar amino acids frequently appear in tandem or pseudo-repeat sequences [63]. Characteristic motifs such as GX, PX, GGX, and PGX—where X typically represents G, A, V, L, or I—are commonly found in these segments [22,64]. Notably, sequences like GV, GGV, and VPG are prevalent in these domains [65]. These repetitive patterns are particularly significant for understanding the structural features of tropoelastin. For example, VPG motifs have been proposed to transiently adopt β-turn conformations, which are believed to contribute to the protein’s dynamic flexibility [66]. In our previous work [24], we examined the structure of (VPGVG)_6_ using various solid-state NMR techniques. Our findings did not support Urry’s β-spiral model, and we estimated that approximately 40% of the structure adopts a type II β-turn conformation.

Regarding the dynamics of water molecules interacting with elastin molecules, two-dimensional relaxation ^2^H NMR techniques were employed [38]. The correlation time, distribution, and population of water in glucose-exposed elastin samples were determined. A particularly valuable aspect of this method is its ability to distinguish between different water reservoirs based on the dynamic behavior of nuclear spins, as resolved on the timescale of the relaxation measurements. While the correlation times of water molecular tumbling were found to be similar across the samples, differences were observed in the relative populations of water.

For the wide use of elastin in biomaterials, soluble elastin is required. Soluble forms of elastin are typically derived from insoluble elastin through hydrolysis. We employed the method developed by Partridge and colleagues with oxalic acid [43,44]. The ^13^C CP/MAS NMR spectra in the dry state indicate that the insoluble spectrum is slightly stiffer than that of the soluble spectrum. Although the difference in the spectrum is small between soluble and insoluble elastin, this slight difference seems to distinguish between solubility and insolubility.

In the dry state, the limited motion of the Val and Leu side chains results in the disappearance of the doublet peaks of Val Cγ and Leu Cδ carbons, which are observed in the hydrated ^13^C solid-state NMR spectra (Figure 5a–c) due to the increased mobility. In addition, the peak intensities of Pro, Val, and Gly residues became smaller in the ^13^C CP/MAS NMR spectrum in the hydrated state than in the dry state (Figure 5c). The ^13^C solid-state NMR chemical shifts of soluble elastin are summarized in Table 1.

### 3.4. ^13^C Solid-State NMR Spectra of Insoluble Elastin in the Dry and Hydrated States

**Figure 6:**
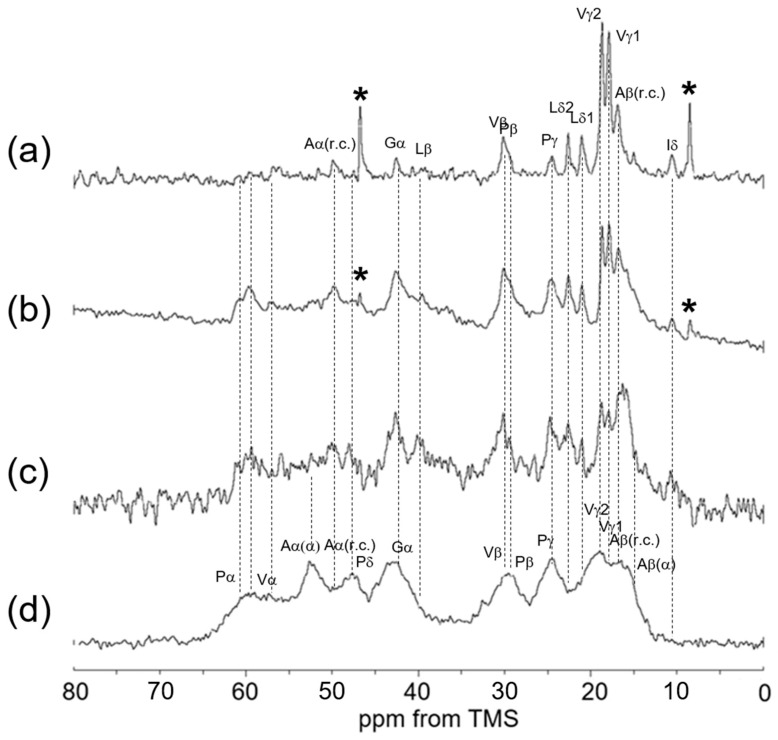
^13^C solid-state NMR spectra (0–80 ppm) of insoluble elastin. (**a**) ^13^C INEPT spectrum, (**b**) ^13^C DD/MAS spectrum, and (**c**) ^13^C CP/MAS spectrum in the hydrated states. (**d**) ^13^C CP/MAS spectrum in the dry state. *: impurity.

Figure 6 shows ^13^C solid-state NMR spectra (0–80 ppm) of insoluble elastin. (a) ^13^C INEPT spectrum, (b) ^13^C DD/MAS spectrum, and (c) ^13^C CP/MAS spectrum in the hydrated states, and (d) ^13^C CP/MAS spectrum in the dry state. The peaks marked by * came from an unidentified impurity. The spectra of the four types of soluble and insoluble elastins are similar to each other. However, the ^13^C CP/MAS NMR spectrum in the hydrated states (Figure 6c) shows that the relative intensities of Gly Cα, Val Cα and Cβ, and Pro Cα, Cβ, Cγ, and Cδ carbons became slightly higher compared to those of these carbons of soluble elastin. This suggests that the hydrophobic domains of insoluble elastin are stiffer than soluble elastin.

### 3.5. Spectral Change of ^13^C Solid-State NMR Spectra of Soluble Elastin in the Dry and Hydrated States Cross-Linked Using Dode-DSP

**Figure 7:**
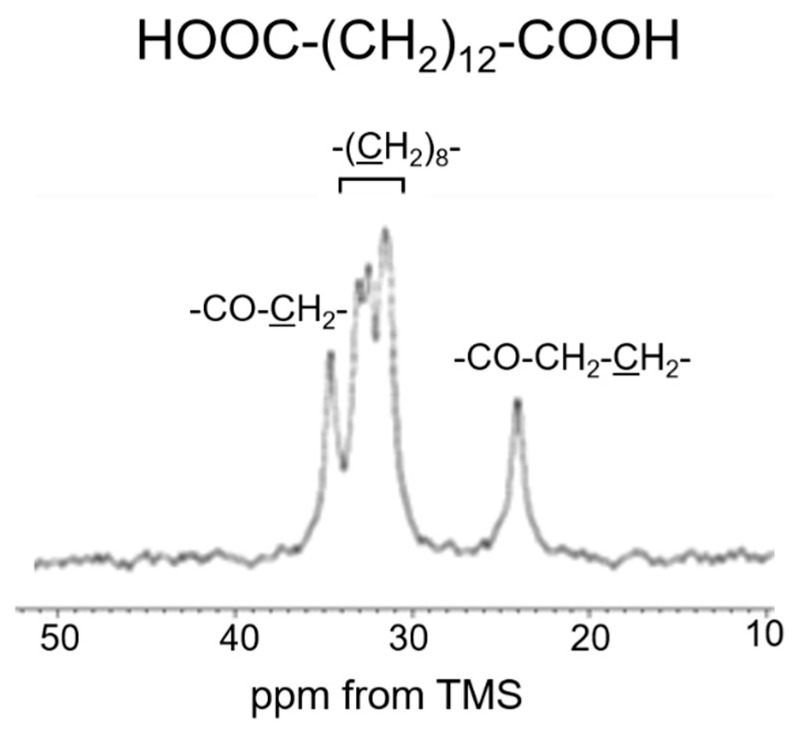
^13^C CP/MAS spectrum of Dode in the dry state.

**Figure 8:**
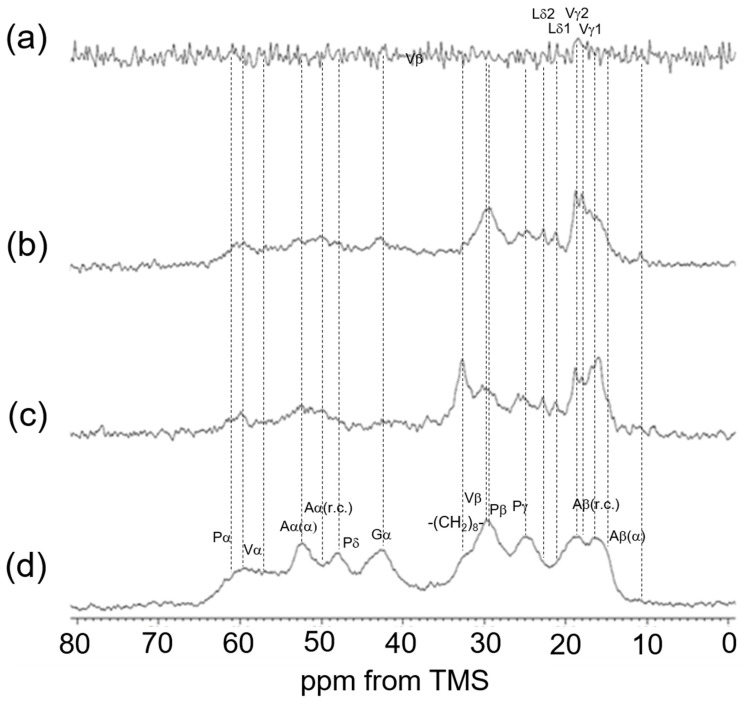
^13^C solid-state NMR spectra (0–80 ppm) of soluble elastin cross-linked by Dode-DSP (×5). (**a**) ^13^C INEPT spectrum, (**b**) ^13^C DD/MAS spectrum, and (**c**) ^13^C CP/MAS spectrum in the hydrated states. (**d**) ^13^C CP/MAS spectrum in the dry state.

**Figure 9:**
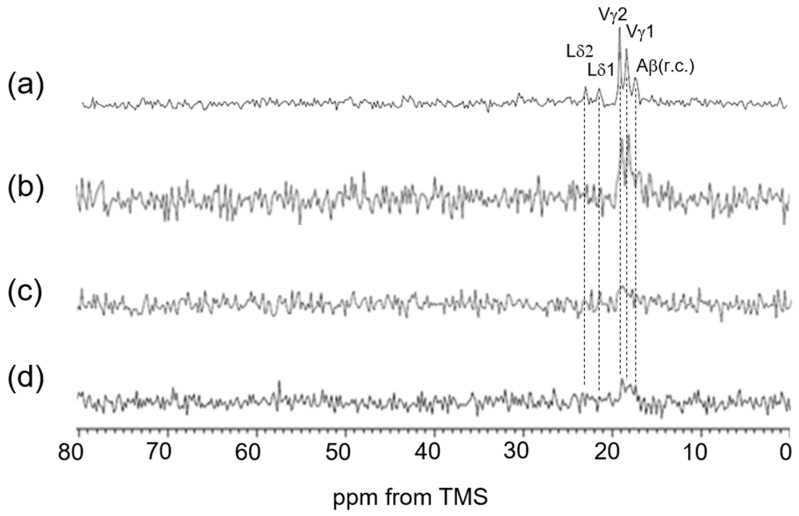
^13^C INEPT spectra in the hydrated states. (**a**) Dode-DSP ×1; (**b**) Dode-DSP ×3; (**c**) Dode-DSP ×5; and (d) Dode-DSP ×7.

**Figure 10:**
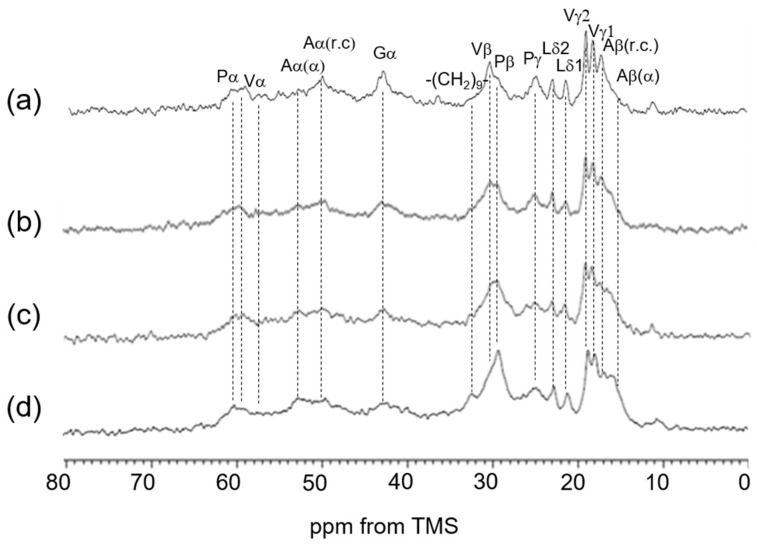
^13^C DD/MAS NMR spectra in the hydrated states. (**a**) Dode-DSP ×1; (**b**) Dode-DSP ×3; (**c**) Dode-DSP ×5; and (**d**) Dode-DSP ×7.

**Figure 11:**
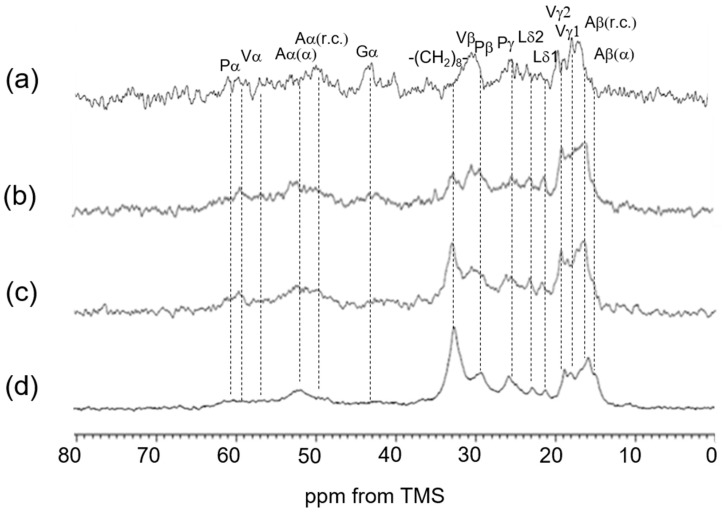
^13^C CP/MAS NMR spectra in the hydrated states. (**a**) Dode-DSP ×1; (**b**) Dode-DSP ×3; (**c**) Dode-DSP ×5; and (**d**) Dode-DSP ×7.

**Figure 12:**
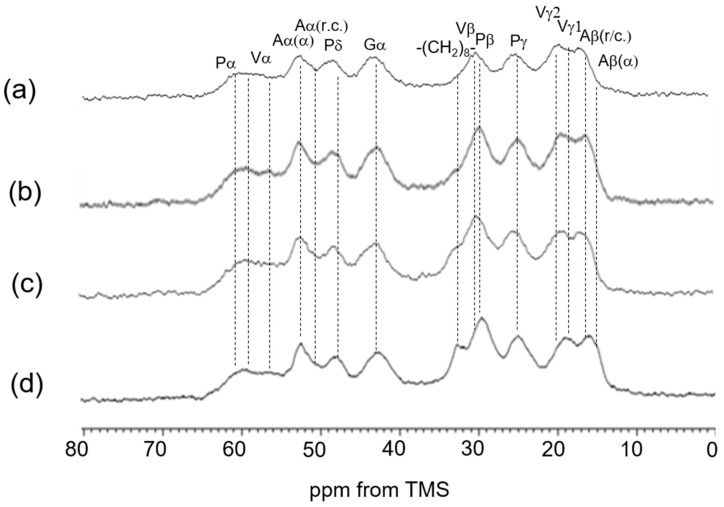
^13^C CP/MAS NMR spectra in the dry states. (**a**) Dode-DSP ×1; (**b**) Dode-DSP ×3; (**c**) Dode-DSP ×5; and (**d**) Dode-DSP ×7.

We also extended our investigation to re-crosslinked soluble elastin treated with the chemical cross-linking agents Dode and DSP [49]. After the cross-linking reaction, Dode molecules remain positioned between the linkers of soluble elastin. Figure 7 shows (a) the ^13^C CP/MAS spectrum of Dode in the dry state. The peaks at 34.6 ppm, 24.1 ppm, and 31.5–32.4 ppm were assigned to -COCH_2_-, -COCH_2_CH_2_- and -(CH_2_)_8_- carbons, respectively. Figure 8 shows ^13^C solid-state NMR spectra (0–80 ppm) of soluble elastin cross-linked by Dode-DSP (crosslinker ratio; ×5), respectively. (a) ^13^C INEPT spectrum, (b) ^13^C DD/MAS spectrum, and (c) ^13^C CP/MAS spectrum in the hydrated states, and (d) ^13^C CP/MAS spectrum in the dry state. Due to further cross-linking formation of soluble elastin by Dode-DSP, the Dode peak was surely observed in the ^13^C CP/MAS NMR spectra of soluble elastin in the hydrated state compared to the dry state. However, only the single -(CH_2_)_8_- peak was detected because the peaks of -COCH_2_- and -COCH_2_CH_2_- carbons shifted due to cross-linking formation in the ^13^C CP/MAS NMR spectra in both dry and hydrated states. A noticeable difference can be observed in the INEPT spectrum when compared to these spectra with the spectra of soluble elastin without cross-linking (Figure 6). Specifically, in the INEPT spectrum of Figure 8a, the ValCγ and Leu Cδ peaks are barely visible. The peak of (CH_2_)_8_ of Dode could be observed at 32.6 ppm in the ^13^C CP/MAS NMR spectra in both the dry and hydrated states (Figure 8c,d) as described above. In addition, the Aα(α) and Aβ(α) peaks seem to be observable together with the Aα(r.c.) and Aβ(r.c.) peaks in the hydrated ^13^C CP/MAS NMR spectrum. This is different from the hydrated ^13^C CP/MAS NMR spectrum of soluble elastin without cross-linking. To clarify these points, the ^13^C solid-state NMR spectra (0–80 ppm) of soluble elastin cross-linked by Dode-DSP were observed under varying crosslinker ratios, as shown in Figure 9 (^13^C INEPT spectrum), Figure 10 (^13^C DD/MAS spectrum), Figure 11 (^13^C CP/MAS spectrum) in the hydrated states, and Figure 12 (^13^C CP/MAS spectrum) in the dry state. With an increasing DSP cross-linking ratio, the Dode peak intensity increased, which is observed in Figure 10, Figure 11 and Figure 12. The mobile components observed in the ^13^C INEPT spectra (Figure 9) decreased, and only small Val Cγ peaks were observed in the spectrum of the ×7 DSP cross-linking ratio. This indicates that the soluble elastin chain became stiffer with increasing DSP cross-linking ratio. Interestingly, the intensity of 52.5 ppm assigned to the α-helical Ala Cα peak of ^13^C CP/MAS NMR spectra in the dry state increased with increasing DSP concentrations from ×1 to ×7. This tendency was also observed for the ^13^C DD/MAS and ^13^C CP/MAS NMR spectra in the hydrated states. As observed previously in Figure 5 and Figure 6, the helical peaks of Ala Cα of initial insoluble and soluble elastins observed in the dry-state ^13^C CP/MAS NMR spectra disappeared in the hydrated-state ^13^C CP/MAS and ^13^C DD/MAS NMR spectra. Therefore, the α-helical Ala residues of the soluble fraction that appeared by cross-linking Dode-DSP treatment in the hydrated states are considered to be newly produced. The intensities of the Cβ peaks of Val and Pro residues in the hydrophobic domain increased, which were observed clearly in the ^13^C DD/MAS NMR spectrum of the soluble elastin cross-linked by Dode-DSP (×7).

Upon cross-linking with the Dode-DSP reagent, the soluble elastin chain became stiffer. This change is clearly reflected in the INEPT spectrum, which shows noticeable differences compared to the spectrum of non-cross-linked soluble elastin. Furthermore, in the hydrated ^13^C CP/MAS NMR spectrum, peaks corresponding to Ala Cα and Ala Cβ in both α-helix and random coil conformations appear to be observable.

Thus, the preparation of soluble elastin from insoluble elastin, followed by cross-linking treatment, appears promising for the development of biomaterials based on elastin [2].

**Figure 13:**
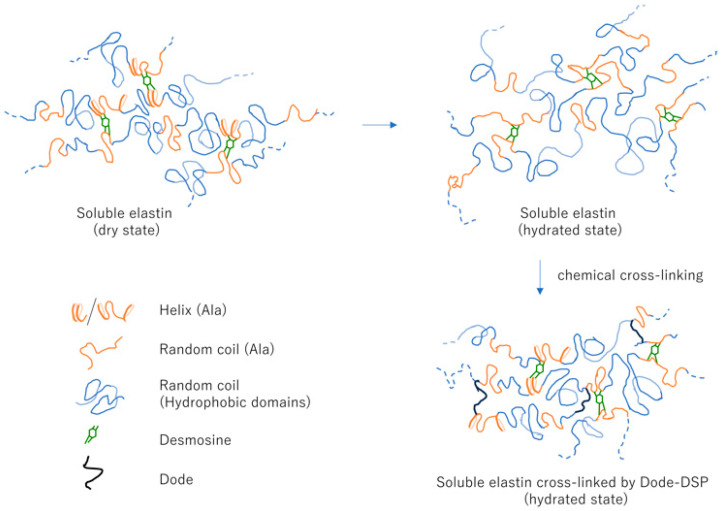
Overview of structural transitions in soluble elastin: dry state, hydrated state, and Dode-DSP re-crosslinked soluble elastin (hydrated state).

Figure 13 is the overview of structural transitions in soluble elastin: dry state, hydrated state, and Dode-DSP re-crosslinked soluble elastin (hydrated state).

Based on the results of this study, the elasticity of elastin is believed to arise from a highly specialized structure that exhibits elastomeric properties. This structure consists of strongly fixed desmosine cross-links and relatively long, flexible polypeptide chains between the cross-linking points. The oxalic acid degradation method, which solubilizes elastin by breaking these robust cross-links, gradually dissolves the protein by cleaving regions other than the cross-linked sites. The resulting soluble elastin (Soluble Elastin (8)), which contains a high amount of desmosine, can be regarded as a water-soluble form that closely resembles insoluble elastin in structure. However, the helical regions near the cleavage sites may become unstable in a hydrated state.

The Dode-DSP used in this study is a cross-linking agent that interacts and aggregates with highly hydrophobic regions. As mentioned earlier, the soluble elastin used in this study contains a high amount of desmosine. Dode-DSP aggregates in the hydrophobic environment surrounding desmosine and selectively reacts with amino-terminal groups near the desmosine cleavage sites, allowing cross-linking without fixing the random and flexible peptide segments between cross-linked regions. In other words, it is considered possible to reconstruct the cross-linked regions near desmosine, which are crucial for the mechanical properties of native elastin. Stabilization of the α-helix is therefore important, and the functional role of desmosine is meaningful as evidence of its structural contribution. This can also be explained paradoxically. Dode-DSP does not react with the free lysine residues in soluble elastin (Soluble Elastin (1)), which contains less desmosine and is eluted early during oxalic acid degradation, and thus cannot be insolubilized—even though it contains more lysine than Soluble Elastin (8). This suggests that Dode-DSP cross-linking occurs only near desmosine structures within hydrophobic environments.

For biomaterials that aim to match the mechanical properties of biological tissues, possessing features similar to the cross-linked structure of native elastin is highly beneficial. Such materials can serve as scaffolds that maintain or reconstruct the dynamic environment of cells and tissues. In fact, the material created by cross-linking the soluble elastin used in this study exhibited an elastic modulus of 50 kPa and an elongation rate of 100%, demonstrating properties similar to those of elastin-rich porcine vascular medial tissue (elastic modulus: 150–200 kPa, elongation rate: 100–150%; original data). As a biomaterial scaffold for vascular endothelial cells and smooth muscle cells, which reside in regions that repeatedly contract and relax due to pulsation and blood pressure regulation, this material is expected to provide compliance with biological tissues and support continuous cellular activity as a valuable non-cellular matrix. Regarding its application, the Dode-DSP crosslinked elastin material is considered useful as a culture substrate or transplantable cell scaffold material that requires dynamic stimulation of cellular activity, since it accurately reproduces the structure and physical properties of natural elastin. It is expected to be beneficial for tissue repair and regenerative medicine materials, including vascular biomaterials, as well as for organs and tissues requiring elasticity, such as the lungs, ligaments, and skin.

In our previous study [67], silk grafts were created by coating this soluble elastin onto knitted silk artificial blood vessels, followed by cross-linking with glutaraldehyde. These silk grafts were then implanted into the abdominal aorta of rats for evaluation. The grafts exhibited patency and remodeling capabilities without adverse reactions such as bleeding during implantation or sutured end disconnection. All grafts remained patent upon extraction, and vascular endothelial cells were observed on the grafts’ luminal surface two weeks post-implantation. Consequently, elastin-coated artificial vascular grafts show potential for applications requiring small-diameter grafts.

### 3.6. Contextualizing the Findings in Biomaterial Design

Collagen and elastin are currently utilized as major bio-derived biomaterials, and materials extracted directly from actual tissues are believed to possess superior cell-responsive functions. However, elastin has a robust cross-linked structure, and when solubilized, this cross-linking is generally disrupted, making it difficult to reproduce its mechanical property of elasticity. This study elucidates the reconstruction of the cross-linked structure through structural analysis and highlights the possibility that the stabilization of alanine helices plays a crucial role in elasticity. Biomaterials require strong interactions with biological systems, and not only the selection of extracellular matrix components but also a precise understanding and demonstration of mechanical properties and structural mimicry are considered essential for designing scaffold materials that regenerate biological tissues.

## 4. Conclusions

Insoluble elastin was extracted from porcine arterial tissue, preserving both its hydrophobic regions and native cross-linked domains. Soluble elastin was then obtained from insoluble elastin through repeated treatments with oxalic acid. To analyze the structure—specifically of the Ala residues in the cross-linking domain—the ^13^C solid-state NMR methods (INEPT, DD/MAS, and CP/MAS NMR) were employed under both dry and hydrated states. In the dry state, α-helix and random coil structures coexisted. However, in the hydrated state, random coil structures became dominant, with partially distorted helix structures in soluble elastin. Furthermore, soluble elastin was cross-linked using the Dode-DSP reagent. In the dry state, Ala residues in the cross-linked elastin exhibited a coexistence of α-helix and random coil structures. Even in the hydrated state, α-helix structures coexisting with random coils were preserved to a greater extent, showing a distinct behavior compared to hydrated soluble elastin without cross-linking treatment. Due to the additional cross-linking formation, the elastin chain became stiffer.

## Figures and Tables

**Table 1:** ^13^C chemical shifts (ppm) and assignments of ^13^C solid-state NMR spectra of soluble elastin.

^13^C Solid-State NMR	Chemical Shifts (ppm)	Assignment	Chemical Shifts (ppm)	Assignment
(a) INEPT (hydrated)	10.5	Iδ	30.2	Vβ
	16.6	Aβ (r.c.)	40.5	Lβ
	17.7	Vγ_2_	42.6	Gα
	18.5	Vγ_1_	48.4	Pδ
	21.0	Lδ_2_	50.0	Aα (r.c.)
	22.6	Lδ_1_	57.0	Vα_2_
	24.3	Lγ	59.7	Vα_1_
	24.7	Pγ	60.9	Pα
	29.5	Pβ		
(b) DD/MAS (hydrated)	10.5	Iδ	42.6	Gα
	16.6	Aβ (r.c.)	48.4	Pδ
	17.8	Vγ_2_	50.0	Aα (r.c.)
	18.7	Vγ_1_	57.0	Vα_2_
	21.1	Lδ_2_	59.4	Vα_1_
	22.5	Lδ_1_	60.8	Pα
	24.6	Pγ + Lγ	129.2	Yγ,δ + Fδ,ε,ζ
	30.1	Pβ + Vβ		
(c) CP/MAS (hydrated)	16.6	Aβ (r.c.)	42.6	Gα
	17.8	Vγ_2_	48.4	Pδ
	18.9	Vγ_1_	50.0	Aα (r.c.)
	21.1	Lδ_2_	57–61	Vα + Pα
	22.7	Lδ_1_	114.9	Yε
	24.6	Pγ	128.6	Yγ,δ + Fδ,ε,ζ
	30.0	Pβ + Vβ	155.0	Yζ
(d) CP/MAS (dried)	15.0	Aβ (α)	50.0	Aα (r.c.)
	16.6	Aβ (r.c.)	52.5	Aα (α)
	18–19	Vγ	57–61	Vα + Pα
	24.6	Pγ	114.9	Yε
	30.0	Pβ + Vβ	128.7	Yγ,δ + Fδ,ε,ζ
	42.6	Gα	155.2	Yζ
	48.4	Pδ		

(r.c.): random coil (α): α-helix. Experimental error: ±0.2 ppm.

## Data Availability

Derived data supporting the findings of this study are available from the corresponding author, T.A., upon request.
